# A clinical scoring system to prioritise investigation for tuberculosis among adults attending HIV clinics in South Africa

**DOI:** 10.1371/journal.pone.0181519

**Published:** 2017-08-03

**Authors:** Yasmeen Hanifa, Katherine L. Fielding, Violet N. Chihota, Lungiswa Adonis, Salome Charalambous, Nicola Foster, Alan Karstaedt, Kerrigan McCarthy, Mark P. Nicol, Nontobeko T. Ndlovu, Edina Sinanovic, Faieza Sahid, Wendy Stevens, Anna Vassall, Gavin J. Churchyard, Alison D. Grant

**Affiliations:** 1 London School of Hygiene & Tropical Medicine, London, United Kingdom; 2 The Aurum Institute, Johannesburg, South Africa; 3 School of Public Health, Faculty of Health Sciences, University of the Witwatersrand, Johannesburg, South Africa; 4 Mamelodi Hospital, Pretoria, South Africa; 5 Health Economics Unit, School of public health and family medicine, University of Cape Town, Cape Town, South Africa; 6 Department of Medicine, Chris Hani Baragwanath Hospital, Johannesburg, South Africa; 7 University of the Witwatersrand, Johannesburg, South Africa; 8 Division of Medical Microbiology, Faculty of Health Sciences, University of Cape Town, Cape Town, South Africa; 9 National Health Laboratory Service, Johannesburg, South Africa; 10 Department of Molecular Medicine and Haematology, School of Pathology, Faculty of Health Sciences, University of the Witwatersrand, Johannesburg, South Africa; 11 Advancing Treatment and Care for TB/HIV, South African Medical Research Council Collaborating Centre for HIV and TB, Johannesburg, South Africa; 12 School of Nursing and Public Health, Africa Health Research Institute, University of KwaZulu-Natal, Durban, South Africa; Katholieke Universiteit Leuven Rega Institute for Medical Research, BELGIUM

## Abstract

**Background:**

The World Health Organization (WHO) recommendation for regular tuberculosis (TB) screening of HIV-positive individuals with Xpert MTB/RIF as the first diagnostic test has major resource implications.

**Objective:**

To develop a diagnostic prediction model for TB, for symptomatic adults attending for routine HIV care, to prioritise TB investigation.

**Design:**

Cohort study exploring a TB testing algorithm.

**Setting:**

HIV clinics, South Africa.

**Participants:**

Representative sample of adult HIV clinic attendees; data from participants reporting ≥1 symptom on the WHO screening tool were split 50:50 to derive, then internally validate, a prediction model.

**Outcome:**

TB, defined as “confirmed” if Xpert MTB/RIF, line probe assay or *M*. *tuberculosis* culture were positive; and “clinical” if TB treatment started without microbiological confirmation, within six months of enrolment.

**Results:**

Overall, 79/2602 (3.0%) participants on ART fulfilled TB case definitions, compared to 65/906 (7.2%) pre-ART. Among 1133/3508 (32.3%) participants screening positive on the WHO tool, 1048 met inclusion criteria for this analysis: 52/515 (10.1%) in the derivation and 58/533 (10.9%) in the validation dataset had TB. Our final model comprised ART status (on ART > 3 months vs. pre-ART or ART < 3 months); body mass index (continuous); CD4 (continuous); number of WHO symptoms (1 vs. >1 symptom). We converted this to a clinical score, using clinically-relevant CD4 and BMI categories. A cut-off score of ≥3 identified those with TB with sensitivity and specificity of 91.8% and 34.3% respectively. If investigation was prioritised for individuals with score of ≥3, 68% (717/1048) symptomatic individuals would be tested, among whom the prevalence of TB would be 14.1% (101/717); 32% (331/1048) of tests would be avoided, but 3% (9/331) with TB would be missed amongst those not tested.

**Conclusion:**

Our clinical score may help prioritise TB investigation among symptomatic individuals.

## Introduction

The World Health Organization (WHO) recommends, as part of activities to address the vast global burden of HIV-related tuberculosis (TB), regular screening for active TB of all people living with HIV (PLHIV) followed by Xpert MTB/RIF (Cepheid, Sunnyvale, CA) as the primary diagnostic test. [1] The recommended TB screening tool, which comprises any one of current cough, fever, weight loss or night sweats (subsequently referred to as the WHO tool), was developed for use in resource limited settings. [2] This simple tool, which was designed to rule out TB prior to the provision of isoniazid preventive therapy (IPT) to PLHIV, maximises sensitivity (78.9%) and negative predictive value (97.7% at TB prevalence of 5% in PLHIV), but has low specificity (49.6%) and positive predictive value (8% at TB prevalence of 5% in PLHIV). [2] South Africa, which is home to the world’s largest HIV epidemic [3] and where 62% of individuals with TB are also HIV-positive, [4] has rolled out Xpert as the initial diagnostic test for all individuals with symptoms suggesting TB. [5] Regular TB screening of PLHIV with a tool that generates large numbers of patients requiring further investigation, of whom only a small proportion will have TB, combined with a diagnostic test that is currently far more expensive than smear microscopy, poses a huge challenge in resource constrained settings. In these settings prioritising testing for those at greatest risk of TB will help preserve resources.

Multivariable prediction models estimate the probability that an individual either has or will develop a particular condition. These models are increasingly abundant in the literature, with variable quality of construction as well as reporting, as highlighted by the recent TRIPOD statement which presents a recommended reporting framework. [6, 7] Clinical scoring algorithms have been developed for PLHIV with symptoms suggestive of TB to prioritise investigation for those with greatest probability of having TB prior to antiretroviral therapy (ART) initiation, [8] and improve case finding, [9] but these algorithms have not been validated or applied to patients on ART.

The aim of our study was to develop a score, comprising elements readily available in primary care, to predict probability of TB in adults attending for routine HIV care screened for TB and found WHO tool positive. This score was used to develop a simple tool to help health care workers in resource limited settings decide whom to prioritise for TB investigation.

## Methods

**Fig 1:**
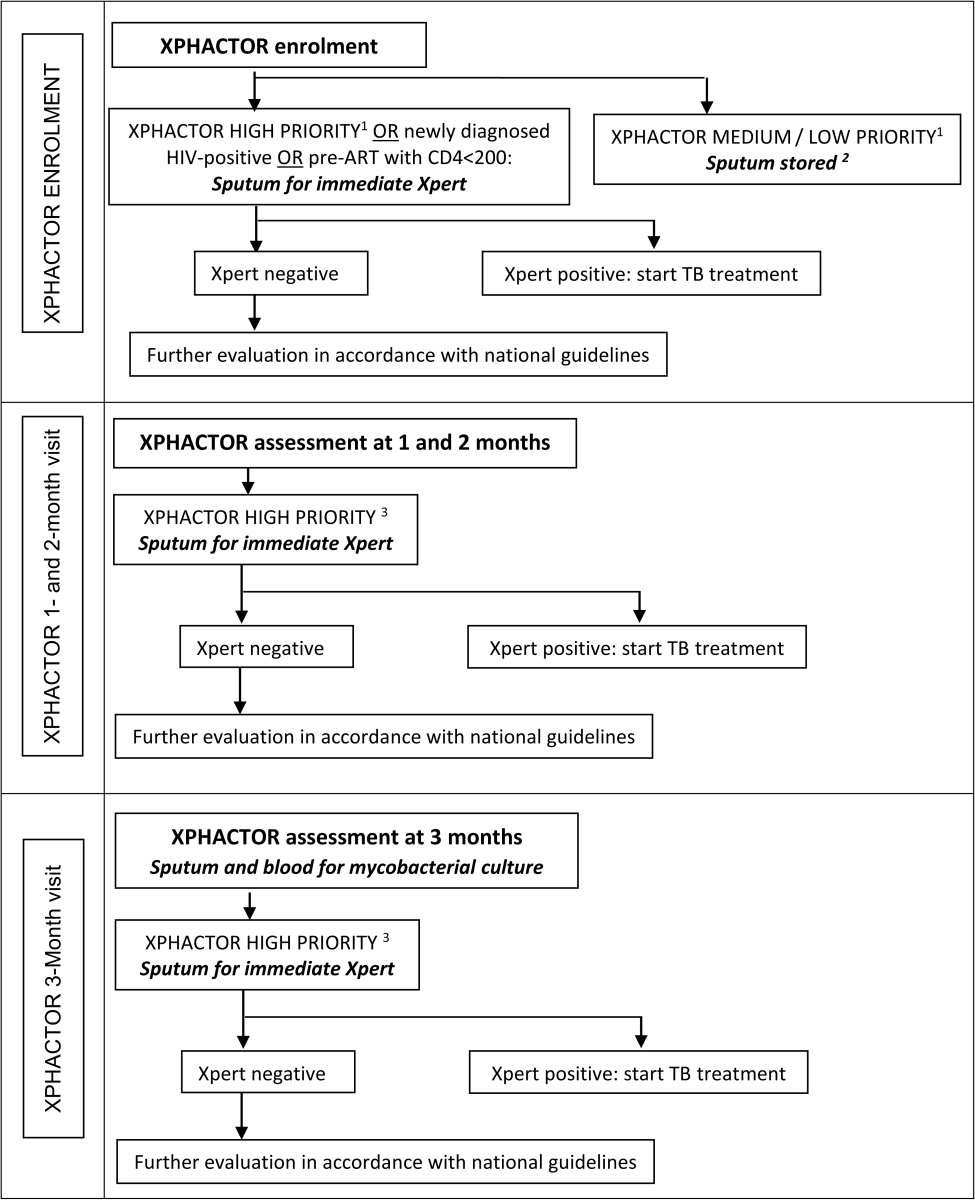
XPHACTOR study flow. ^1^ High priority (any of: current cough, fever ≥ 3 weeks, body mass index (BMI) <18.5 kg/m^2^, CD4 <100x10^6^/l, measured weight loss ≥10% in preceding 6 months, or other feature raising high clinical suspicion of TB); medium priority (any of: fever < 3 weeks, night sweats, measured weight loss <10% in preceding 6 months); low priority = no TB symptoms. ^2^ Samples tested with Xpert MTB/RIF at the end of the study. ^3^ High priority (any of: current cough, fever ≥ 3 weeks, night sweats ≥ 4 weeks, body mass index (BMI) <18.5 kg/m^2^, CD4 <100x10^6^/l, measured weight loss ≥10% in preceding 6 months, or other feature raising high clinical suspicion of TB); medium priority (any of: fever < 3 weeks, night sweats <4 weeks, measured weight loss <10% in preceding 6 months); low priority = no TB symptoms.

We used data collected for “Xpert for people attending HIV/AIDS care: test or review?” (XPHACTOR), a prospective cohort study evaluating a risk-based algorithm to prioritise Xpert MTB/RIF testing amongst adults attending for routine HIV care in South Africa, to develop and validate our clinical score. Fig 1 depicts XPHACTOR study flow.

### XPHACTOR study population and recruitment

We enrolled a systematic sample of adults (aged ≥18 years) attending two hospital-based and two community health centre (CHC) clinics in Gauteng province, South Africa, for HIV care, irrespective of presence of symptoms suggestive of TB. Patients taking anti-tuberculosis treatment within the previous 3 months were excluded. Patients were enrolled into three groups: “on antiretroviral therapy (ART)” (currently taking or ART-experienced) group; “pre-ART” (in HIV care but not yet taking ART) group; and “HIV Testing and Counselling (HTC)” (newly-diagnosed HIV-positive). We recruited to the on ART group from hospital clinics because their patient population solely comprised those ART-experienced; and pre-ART and HTC groups were recruited from CHC clinics. At the time of the study, ART eligibility comprised CD4 ≤350 cells/mm^3^ or WHO clinical stage ≥3.

### XPHACTOR procedures

#### Enrolment

At enrolment, research staff administered a standardised questionnaire incorporating the WHO TB screening tool (any of current cough, fever, night sweats or unintentional weight loss), measured height and weight, mid-upper arm circumference (MUAC), and recorded most recent clinic CD4 cell count. Further investigation was prioritised according to the XPHACTOR algorithm with an immediate spot sputum sample sent for Xpert MTB/RIF for (i) all assigned “high priority” (any of: current cough, fever ≥ 3 weeks, body mass index [BMI] <18.5 kg/m^2^, CD4 <100x10^6^/l, measured weight loss ≥10% in preceding 6 months, or other feature raising high clinical suspicion of TB); (ii) those in pre-ART group with CD4<200 x10^6^/l at enrolment (iii) all in HTC group at enrolment, the latter two categories (who were recruited for XPHACTOR substudies) because of *a priori* high risk of active TB. For all other participants a spot sputum sample was frozen at -80°C within 24 hours, for testing with Xpert at the end of the study.

#### Follow-up

Participants were reviewed monthly to three months, with repeat WHO symptom screen and a spot sputum requested for Xpert MTB/RIF if “high priority” by the study algorithm at that visit, with the exception of those in the “on ART” group who were asymptomatic at enrolment who were telephoned at 1 and 2 months to update locator information but were not asked about TB symptoms. At the 3-month visit sputum (induced if necessary) and blood were collected for mycobacterial culture on liquid media (Bactec MGIT 960 and 9240 systems) from all study participants. We allowed a broad window period around the scheduled 3-month visit, till around six months, in order to maximise study follow-up.

Participants who submitted an Xpert sample were reviewed and if Xpert-positive, TB treatment was initiated; if negative, further investigation in accordance with national guidelines was facilitated (chest radiograph, sputum culture and trial of antibiotics).

Results of all investigations were fed back to clinic staff, who were responsible for management decisions. Clinic medical records were reviewed at the end of the study to ascertain any additional relevant investigations and/or TB diagnoses. Deaths were identified through reports from participant-nominated contacts, clinic staff, and by accessing the Department of Home Affairs vital statistics database using participants’ South African identification (ID) numbers.

### Development and validation of the prediction model

#### Participants

We restricted our analysis to all XPHACTOR participants who were WHO tool positive at enrolment and established in care (i.e. not newly testing HIV positive); and excluded those taking isoniazid preventive therapy (IPT) at enrolment, as those on IPT were likely to have recently undergone investigation for TB, and hence were effectively “pre-screened” for TB.

To be deemed clinically useful a prediction model should demonstrate accurate prediction of the outcome in data other than that in which the model was developed. We developed our prediction model using part of our dataset, and undertook internal validation of model performance using the remainder of the dataset. [10] Enrolment to XPHACTOR was staggered by site, commencing with hospital clinics; hence the dataset was stratified by site and split 50:50 by median date of enrolment within site. Data from the earlier half were used to derive our prediction model (derivation dataset), and from the latter half for validation (validation dataset). Data were analysed using Stata 14 (Stata Corporation, College Station, TX, USA), as detailed below.

#### Outcome

Our outcome was confirmed or clinical TB versus “not TB”, ascertained within 6 months of enrolment to XPHACTOR, as defined below.

“Confirmed” TB was defined as a positive result on i) Xpert MTB/RIF or ii) line probe assay (LPA) performed on smear-positive or cultured isolate (GenoType MTBDR*plus*, Hain Lifesciences*)* or iii) *M*. *tuberculosis* (*Mtb*) culture, from any sample (including stored sputum and those requested by the health care provider) collected within six months of XPHACTOR enrolment. Individuals who started TB treatment within six months of enrolment (including those with treatment starts reported in the context of a separate verbal autopsy sub-study), in the absence of microbiological confirmation, were assigned “clinical” TB. This was based on the assumption that an HIV-positive adult with a positive bacteriological test result or starting TB treatment within six months after enrolment likely had active TB at enrolment, supported by data from Zimbabwe which estimated the mean duration of smear-positivity prior to TB diagnosis amongst HIV-positive adults to be 18–33 weeks. [11]

“Not TB” was defined as fulfilling all of the following: absence of criteria for confirmed or clinical TB; and alive at least 3 months after enrolment. Participants who did not fulfil the case definitions for TB or “not TB” were deemed to have an unclassifiable outcome and excluded from the analyses.

Pulmonary and extrapulmonary TB were classified in accordance with WHO definitions. [12]

#### Candidate predictor selection

There is no consensus on the best method for selecting candidate variables, but suggested approaches include using literature review, clinical knowledge and studying the distribution of predictors in the study data. [6, 13, 14] It is recommended, to ensure predictive accuracy, that the total number of candidate predictors is limited so that there are at least 10 outcomes for each candidate predictor studied. [6, 13] We considered predictors from data collected at enrolment to XPHACTOR known to be associated with prevalent and/or incident TB amongst PLHIV: age, sex, previous TB treatment, smoking, alcohol use, history of ART, duration on ART, previous IPT, previous cotrimoxazole preventive therapy (CPT), presence of individual WHO tool symptoms, duration of WHO tool symptoms, BMI, MUAC, CD4 count, haemoglobin, and viral load. [15–25] History of mining, [26] health care work, [27] and incarceration, [28] although established risk factors for TB were not considered as <10% participants fell into each category. The following variables were also excluded: MUAC, measured weight loss, haemoglobin and viral load, due to >20% missing data; and previous IPT, as there was only one outcome amongst participants with previous IPT.

*A priori* we combined history of ART with duration on ART to generate “ART status” categorised as: pre-ART or on ART <3 months *vs*. on ART for >3 months, as amongst patients on ART, duration of <3 months is a predictor for prevalent TB. [29] *A priori* we considered ART status, CD4 cell count, and BMI for our adjusted model, and used univariable screening to select additional candidate predictors with P-value (p)<0.25.

#### Model building procedures in derivation dataset

We undertook multivariable logistic regression of candidate predictors, sequentially removing the variable with the largest Wald *p*-value >0.05 (stepwise backward elimination), to generate our final model. [13] A complete-case analysis was undertaken, excluding participants with missing information relating to any of the candidate predictors. A model that categorised the number of WHO symptoms as 1 vs. >1 symptom (model A) was compared with one that included individual WHO tool symptoms (model B), aiming to select the simplest and most practical model to implement in primary care. We also considered a model without CD4 count for settings where this might not be easily available.

Transformations of continuous variables (BMI and CD4) were assessed using fractional polynomials. In our final selected model we tested for interactions between remaining variables and “ART status”.

#### Assessing model performance in derivation dataset

We assessed model calibration, the agreement between probability of TB predicted by the model and observed probability of TB within quantiles of predicted risk, graphically in a calibration plot; and statistically using the Hosmer-Lemeshow test. We assumed p<0.05 from the Hosmer-Lemeshow test as indicating lack of model fit (poor calibration), although the test has limited statistical power to detect poor calibration unless the sample size is large and the outcome frequent. [13] We assessed discrimination, the ability of our model to differentiate patients with TB vs. those without, using the area under the receiver-operating characteristic curve (AUROC). AUROC 0.7 to 0.79, 0.8–0.89, ≥0.9 are respectively considered acceptable, excellent and outstanding discrimination. [30]

### Transformation from regression model to clinical score in derivation dataset

Continuous variables in the final model were categorised in a clinically meaningful manner based on their functional form, and each beta coefficient from this logistic regression model was divided by the smallest coefficient and rounded to the nearest integer to assign points to each variable. The total number of points was summed for each participant to calculate the clinical score.

#### Internal validation

We used the beta coefficients and intercept from the final regression models (before and after categorisation of continuous variables) generated from the derivation dataset to calculate the risk score for each participant in our validation dataset. We converted the risk score into predicted risk using predicted risk = 1/(1+e^-risk score^), [13] and assessed performance of the regression model in the validation dataset by evaluating calibration and discrimination.

### Ethical approval

The study was approved by the ethics committees at the University of the Witwatersrand, University of Cape Town, and the London School of Hygiene & Tropical Medicine. All consenting participants gave written consent or, for illiterate participants, witnessed verbal consent. For illiterate participants, there was an impartial witness present during the consenting process, who then signed the relevant witness section of the consent form. All ethics committees approved the consent form, including the section on the use of witnessed oral consent for illiterate participants, at the beginning of the study. Principles expressed in the Declaration of Helsinki were followed in the conduct of this research.

## Results

**Fig 2:**
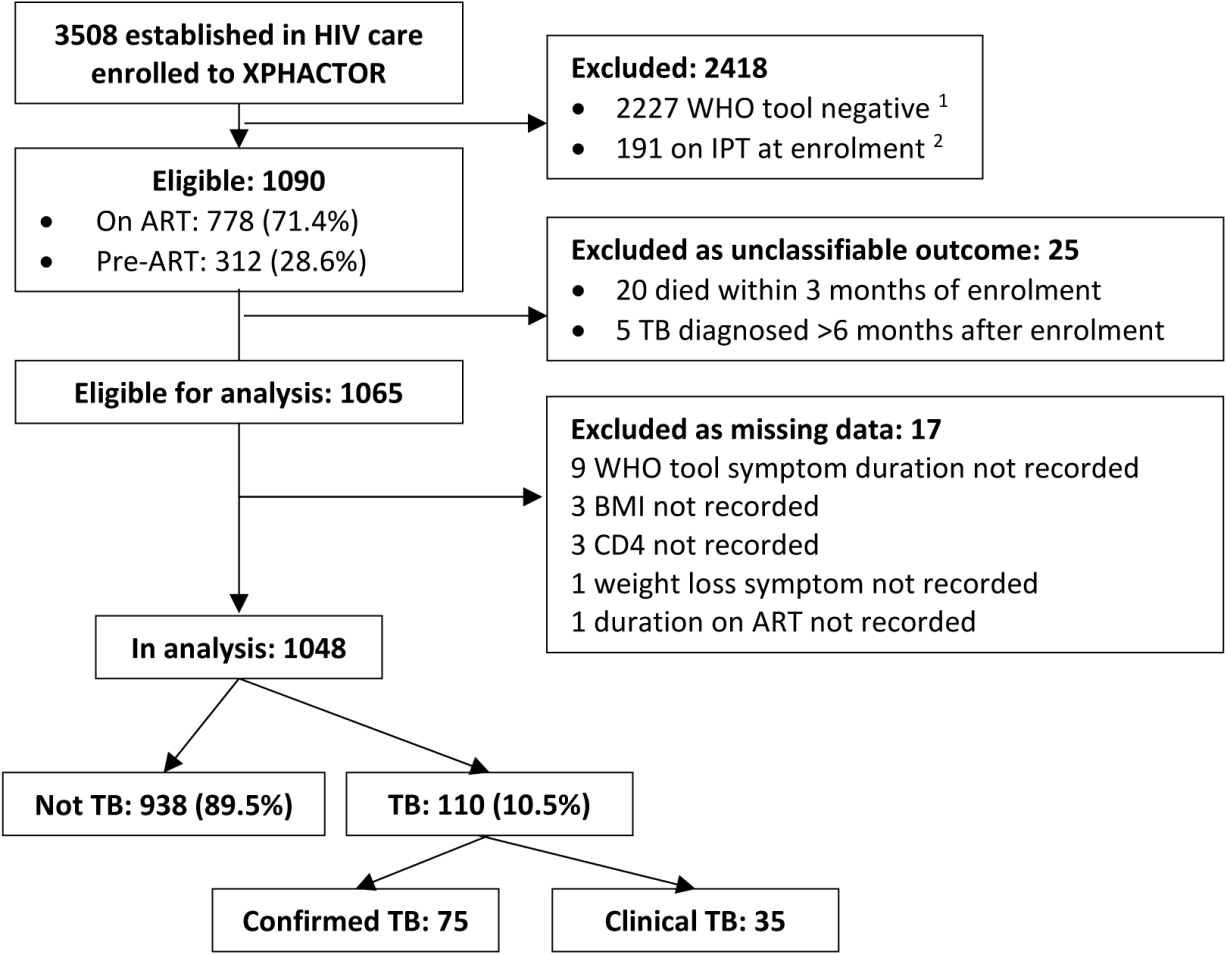
Flow chart of study participants. ^1^ 28/2227 TB diagnosed within six months of enrolment (25 confirmed TB and 3 clinical TB), of whom 25 on ART and 3 pre-ART. ^2^ 4/191 confirmed TB diagnosed within six months of enrolment, all pre-ART BMI = body mass index. IPT = Isoniazid preventive therapy. WHO tool negative = self-report of absence of all of: current cough, fever, night sweats and unintentional weight loss.

We enrolled 3508 participants established in care (i.e. not newly testing HIV positive) to XPHACTOR. Overall, among patients taking ART, 783/2602 (30.1%) reported one or more symptom in the WHO tool and 79/2602 (3.0%) had TB. Among pre-ART patients 350/906 (38.6%) reported ≥1 symptom and 65/906 (7.2%) had TB. For this analysis, 2418/3508 were excluded because WHO tool negative (2227) or on IPT at enrolment (191), and a further 25 participants were excluded because of “unclassifiable” outcome leaving 1065 who were WHO tool positive and eligible for our analysis (Fig 2). We undertook a complete-case analysis and therefore excluded a further 17 participants with missing candidate predictor data (S1 Table), leaving 1048 for our analysis.

### Characteristics of study participants

Table 1 compares the characteristics of participants in the derivation and validation datasets. There were 515 participants in the derivation dataset, enrolled between September 2012 and September 2013, amongst whom 52 (10.1%) participants fulfilled case definitions for TB (36 confirmed, 16 clinical). In the validation dataset there were 533 participants enrolled between May 2013 and March 2014, amongst whom 58 (10.9%) participants fulfilled case definitions for TB (39 confirmed, 19 clinical). The proportion with pulmonary vs. extrapulmonary disease in derivation vs. validation datasets amongst those with confirmed TB was pulmonary (35/36 vs. 37/39) and extrapulmonary (1/36 vs. 2/39); and amongst those with clinical TB was pulmonary (9/16 vs. 7/19), extrapulmonary (4/16 vs. 5/19) and not recorded (3/16 vs. 7/19). [12] The median time from enrolment to earliest of positive TB test or date TB treatment was started amongst all participants diagnosed with TB (derivation and validation datasets combined) was 7 days (IQR 0, 63), with 90% of diagnoses made within 120 days of enrolment.

**Table 1:** Characteristics of participants in derivation and validation datasets.

Characteristic		Derivation dataset (N = 515)	Validation dataset (N = 533)
		Value	Value
		N (%)	N (%)
**Demographics**			
**Age, years**	Median (IQR)	41 (34,48)	41 (34,48)
**Sex**	Female	345 (67.0%)	377 (70.7%)
**Alcohol history**	Never ^1^	308 (59.8%)	353 (66.2%)
**Smoking history**	Never ^2^	354 (68.7%)	383 (71.9%)
**HIV/TB history**			
**Participant category**	On ART ^3^	370 (71.8%)	381 (71.5%)
**Duration since HIV diagnosed, months**	Median (IQR)	56 (21,95) (N = 505)	51 (6,97) (N = 531)
**Duration on ART, months**	Median (IQR)	55 (26,85) (N = 370)	51 (28,83) (N = 381)
**Ever had IPT**	Yes	51 (9.9%)	19 (3.6%)
**Ever had CPT**	Yes	370 (71.8%)	355 (66.6%)
**Previous TB treatment**	Yes	201 (39.0%)	199 (37.3%)
**WHO symptoms at enrolment**			
	Cough	304 (59.0%)	350 (65.7%)
	Weight loss	235 (45.6%)	221 (41.5%)
	Night sweats	131 (25.4%)	130 (24.4%)
	Fever	97 (18.8%)	88 (16.5%)
**Number of symptoms**	1	341 (66.2%)	352 (66.0%)
	2	114 (22.1%)	122 (22.9%)
	3	42 (8.2%)	43 (8.1%)
	4	18 (3.5%)	16 (3.0%)
**Duration of WHO symptoms**^4^, **days**	Median (IQR)	30 (8,89)	28 (7,84)
**CD4 / BMI at enrolment**			
**CD4, cells/mm**^**3**^	Median (IQR)	378 (228,543)	334 (168,559)
	Range	1–1630	2–1577
**Time from CD4 to enrolment, days**	Median (IQR)	147 (45,259)	116 (27,267) (N = 528)
**BMI, kg/m**^**2**^	Median (IQR)	24.0 (20.6,28.4)	24.0 (20.2,28.4)
	Range	13.4–47.2	15.0–57.9
**TB diagnoses over 6 months follow-up**			
	Total	52 (10.1%)	58 (10.9%)
	Confirmed TB	36 (7.0%)	39 (7.3%)
	Clinical TB	16 (3.1%)	19 (3.6%)
**Time from enrolment to TB diagnosis** ^**5**^, **days**	Median (IQR)	7 (0,31) (N = 52)	8 (0,83) (N = 57)
**Follow up**			
**Time from enrolment to most recent of last study / clinic** ^**6**^ **visit**, **days**	Median (IQR)	280 (203,350) (N = 514)	179 (133,231) (N = 532)
**Alive 6 months after enrolment** ^**7**^	Yes	98% (477/487)	98% (463/473)

^1^ compared with any alcohol in last 1 year

^2^ compared with ever/ex-smoker

^3^ compared with pre-ART group

^4^ duration WHO tool positive

^5^ defined as earliest of positive TB test or date TB treatment started

^6^ Most recent clinic visit at time of clinic file review

^7^ Amongst participants with most recent study/clinic visit <6 months from enrolment, if participant had valid South African ID number and demise not reported by Department of home affairs / participant-nominated contacts / clinic staff within 6 months of enrolment, participant assumed to be alive at 6 months after enrolment.

IPT = isoniazid preventive therapy; CPT = cotrimoxazole preventive therapy; IQR = interquartile range

In derivation and validation datasets, median age was 41 years, 72% were in the on ART group, most participants were female (67% vs. 71%), and the most common WHO tool symptoms reported at enrolment were cough (59% vs. 66%) and weight loss (46% vs. 42%; Table 1). At enrolment median CD4 was greater in derivation compared with validation dataset (378 vs. 334 cells/mm^3^), and median BMI was similar (24 kg/m^2^). Participants in the derivation dataset were more likely to report previous IPT than those in the validation dataset (9.9% vs. 3.6%).

### Development of regression model (derivation dataset)

**Fig 3:**
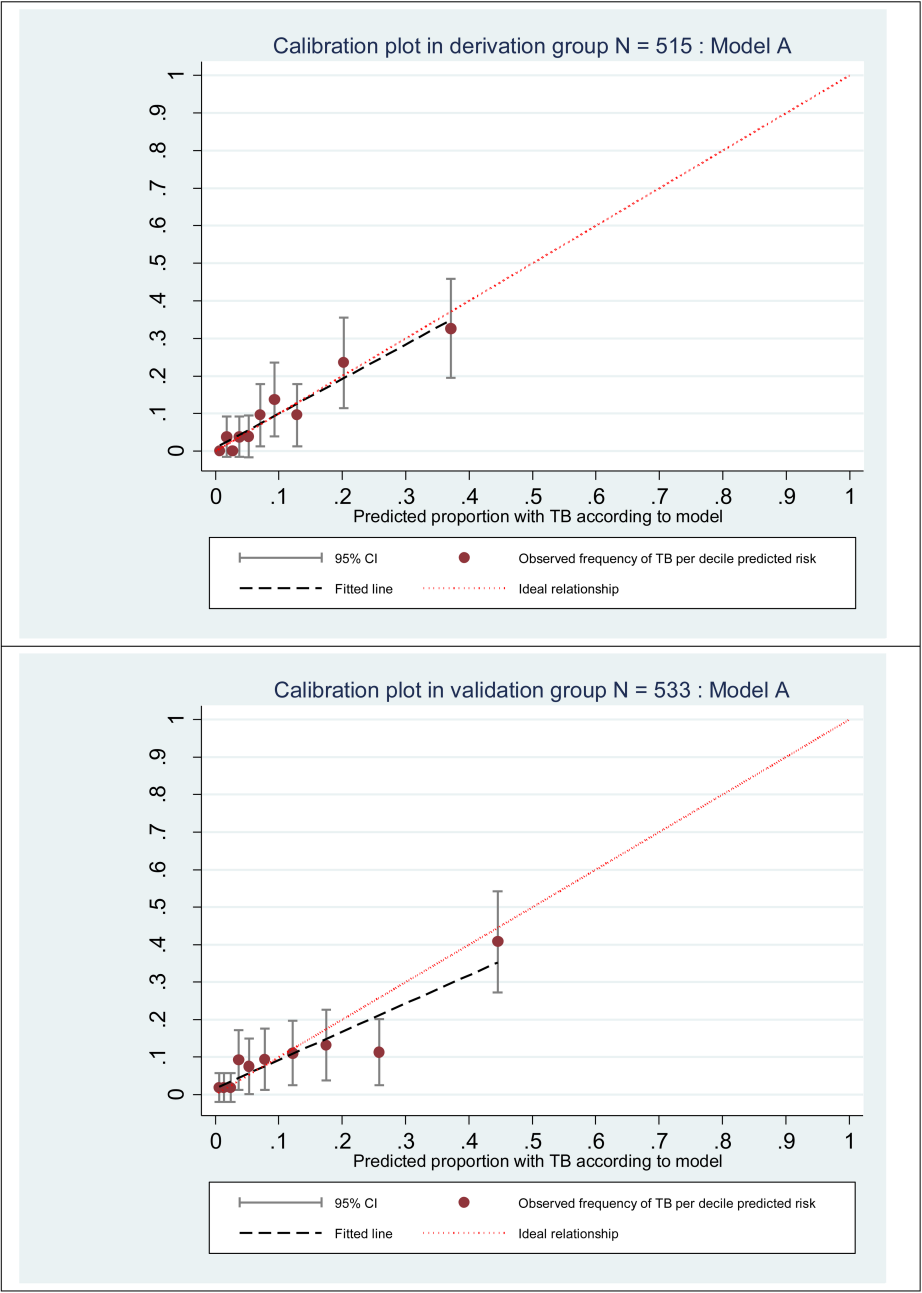
Calibration plot of final prediction model in derivation and validation datasets.

Table 2 summarises the candidate predictors considered for model A, which categorised number of WHO symptoms reported (1 symptom vs. > 1 symptom), and the final multivariable model. We excluded age, alcohol status and previous history of TB as p>0.25 in univariable analysis. Our final model (model A) comprised: ART status (on ART > 3 months = 0 *v* pre-ART or ART <3 months = 1); BMI (continuous, linear); CD4 (continuous, linear); number of WHO symptoms (1 symptom = 0 *v* >1 symptom = 1). A linear relationship with log odds of the outcome was found to be adequate using fractional polynomials for both BMI and CD4 count. No evidence was found for statistical interactions between ART status and CD4 count, BMI, or number of WHO symptoms (Wald p-value ≥0.9). This model had Hosmer-Lemeshow statistic p = 0.65 and AUROC 0.79 (95% confidence intervals [CI] 0.73–0.86) indicating statistically adequate calibration and discrimination in the derivation dataset (Fig 3, S2 Table). In a sensitivity analysis where we excluded all clinical TB and used a gold standard of bacteriologically-confirmed TB, we obtained the same final multivariable model (S3 Table).

**Table 2:** Univariable and multivariable logistic regression analysis in the derivation dataset (N = 515).

Predictor		Patients with TB	Unadjusted	P value	Adjusted ^3^	P value	Adjusted β
		N = 52/515	odds ratio	(Wald)	odds ratio		coefficient
		n/N (%)	(95% CI)		(95% CI) Model A		(log [adjusted OR])
							(95% CI)
**Age ^1^, years**			1.00 (0.97, 1.03)	0.96			
**Sex**	Male	23/170 (13.5%)	1				
	Female	29/345 (8.4%)	0.59 (0.32, 1.05)	***0*.*07***			
**Smoking status**	Never smoked	28/354 (7.9%)	1				
	Current or ex-smoker	24/161 (14.9%)	2.04 (1.14, 3.64)	***0*.*02***			
**Alcohol status**	Current	23/207 (11.1%)	1				
	None in last 1 year	29/308 (9.4%)	0.83 (0.47, 1.48)	0.53			
**ART status**	On ART ≥ 3 months	24/347 (6.9%)	1		1		0
	Pre-ART / ART <3 months	28/168 (16.7%)	2.69 (1.51, 4.80)	***0*.*001***	2.22 (1.17, 4.22)	***0*.*01***	0.80 (0.16, 1.44)
**Ever had CPT**	No / don’t know	19/145 (13.1%)	1				
	Yes	33/370 (8.9%)	0.65 (0.36, 1.18)	***0*.*16***			
**Previous history of TB**	No	33/314 (10.5%)	1				
	Yes	19/201 (9.5%)	0.89 (0.49, 1.61)	0.70			
**Number of WHO symptoms**	1 symptom	18/341 (5.3%)	1		1		0
	> 1 symptom	34/174 (19.5%)	4.36 (2.38, 7.98)	***<0*.*001***	**3.45 (1.83, 6.49)**	***<0*.*001***	1.24 (0.60, 1.87)
**Duration of WHO tool symptoms**	<1 week	3/97 (3.1%)	1				
	≥ 1 week	49/418 (11.7%)	4.16 (1.27, 13.64)	***0*.*02***			
**BMI ^1^^,^^2^, kg/m^2^**			0.88 (0.82, 0.94)	***<0*.*001***	0.89 (0.83, 0.95)	***0*.*001***	-0.12 (-0.19, -0.05)
**CD4 ^1^^,^^2^, cells/mm^3^**			0.997 (0.995, 0.998)	***<0*.*001***	0.998 (0.996, 0.999)	***0*.*006***	-0.002 (-0.004, -0.0006)

^1^ Age, BMI and CD4 count were modelled as continuous variables

^2^ In the multivariable analysis BMI and CD4 count were modelled as continuous variables, a linear relationship with log odds of outcome was found to be adequate after modelling using fractional polynomials.

^3^ Adjusted for all variables shown in column. 100 unit increase in CD4 corresponds to reduction in adjusted odds ratio (aOR) of TB of 0.80 (95% CI 0.68, 0.94); 5 unit increase in BMI corresponds to reduction in aOR of TB of 0.56 (95% CI 0.39, 0.79).

Intercept (log odds) for multivariable model is 0.39.

In the validation dataset the risk score was calculated using the formula: risk score = 0.39 + 0.80 (if pre-ART / ART< 3months)–(0.002 x CD4 count)–(0.12 x BMI) + 1.24 (if > 1 symptom)

Univariable screening to select candidate predictors may result in the rejection of important predictors. [6, 13] When we repeated our multivariable analysis without univariable screening, and included all candidate predictors considered for model A, using stepwise backward elimination we obtained the same final model.

### Internal validation of final regression model

The risk score and predicted risk were calculated for the validation dataset using model A and showed that calibration and discrimination were adequate (Hosmer-Lemeshow p = 0.31 [S2 Table], AUROC 0.75 [95% CI 0.68–0.82]), though the calibration plot demonstrates over-prediction at higher deciles of risk (Fig 3).

### Alternative prediction models

S4 Table presents an alternative multivariable model developed using individual WHO tool symptoms rather than total number of symptoms (model B). Model B comprised ART status, BMI, cough, night sweats and unintentional weight loss. In the derivation dataset there was evidence that presence of cough was modified by ART status (p = 0.03 for interaction term); and the model had adequate calibration and discrimination (Hosmer-Lemeshow statistic p = 0.81, AUROC 0.82 [95% CI 0.76–0.88]). In the validation dataset this model had poor calibration (Hosmer-Lemeshow statistic p = 0.01) although discrimination was acceptable (AUROC 0.75 [95% CI 0.69–0.82]).

We repeated our multivariable analysis using all candidate predictors considered for model A removing CD4 count, for use in a setting where CD4 count is not easily obtainable. This model containing ART status, BMI and number of WHO symptoms (data not shown), performed adequately in the derivation dataset (Hosmer-Lemeshow statistic p = 0.54, AUROC 0.77 [95% CI 0.71–0.84]). In the validation dataset this model had poor calibration (Hosmer-Lemeshow statistic p = 0.02) although discrimination was acceptable (AUROC 0.70 [95% CI 0.63–0.77]).

We selected model A as our final model to develop the risk score because it was simpler and performed better in the validation dataset.

### Transformation from regression model to clinical score

We used WHO BMI categorisation of <18.5 kg/m^2^ as underweight, 18.5–24.9 kg/m^2^ as normal weight, and ≥ 25 kg/m^2^ as overweight. CD4 count was categorised as <200 cells/mm^3^, 200–349 cells/mm^3^ and ≥ 350 cells/mm^3^ to reflect clinically relevant cut-offs and the skewed CD4 count distribution amongst HIV-infected patients with TB. [31] The multivariable model with categorisation of these continuous variables in the derivation dataset is presented in Table 3. This model had statistically adequate discrimination in both derivation (AUROC 0.79 [95% CI 0.73, 0.86]) and validation datasets (AUROC 0.72 [95% CI 0.65, 0.79]). The Hosmer-Lemeshow statistic *p*-value was 0.89 in the derivation dataset but 0.02 in the validation dataset indicating poor calibration in the validation dataset.

**Table 3:** Multivariable model and clinical score in the derivation dataset (N = 515).

Predictor		Adjusted odds ratio (95% CI)	β coefficient	P value	Score*
**ART status**	On ART ≥ 3 months	1	0		0
	Pre-ART / ART < 3 months	2.34 (1.22,4.46)	0.85	0.01	3
**BMI, kg/m^2^**	≥ 25	1	0		0
	18.5–24.9	2.23 (1.05,4.74)	0.80	0.04	2
	<18.5	6.79 (2.61,17.62)	1.91	<0.0001	6
**CD4, cells/mm^3^**	≥ 350	1	0		0
	200–349	1.40 (0.63,3.11)	0.34	0.4	1
	< 200	2.55 (1.23,5.30)	0.94	0.01	3
**Number of WHO symptoms reported**	1 symptom	1	0		0
	> 1 symptom	3.59 (1.90,6.80)	1.28	<0.0001	4
**Intercept (log odds)**	-4.23				

* Each coefficient was divided by 0.335 (the smallest coefficient in our model, CD4 200–349 cells/mm^3^) and rounded to the nearest integer to form the score for that predictor

The clinical score for each predictor was generated and the possible range for the total score was 0 to 16 (Table 3).

Table 4 shows the percentage of patients diagnosed with TB at each value of clinical score in derivation and validation datasets, and S1 Fig boxplot illustrates the distribution of clinical score, stratified by dataset, amongst those diagnosed with TB vs. those not diagnosed with TB.

**Table 4:** Performance of clinical score in derivation and validation datasets.

Clinical score	Derivation dataset		Validation dataset	
	Total with score	Number diagnosed with TB (%) ^1^	Total with score	Number diagnosed with TB (%) ^1^
**0**	69	1 (1.5)	90	2 (2.2)
**1**	24	0	32	1 (3.1)
**2**	62	2 (3.2)	54	3 (5.6)
**3**	74	3 (4.1)	50	7 (14)
**4**	33	3 (9.1)	37	3 (8.1)
**5**	52	3 (5.8)	42	2 (4.8)
**6**	54	4 (7.4)	54	2 (3.7)
**7**	41	6 (14.6)	21	4 (19.1)
**8**	23	6 (26.1)	33	6 (18.2)
**9**	21	2 (9.5)	25	2 (8.0)
**10**	27	7 (25.9)	34	4 (11.8)
**11**	7	1 (14.3)	4	1 (25)
**12**	18	8 (44.4)	41	18 (43.9)
**13**	7	4 (57.1)	6	0
**14**	0		2	0
**16**	3	2 (66.7)	8	3 (37.5)
**TOTAL**	**515**	**52**	**533**	**58**

^1^ Row percentages shown

#### Selection of cut-off for clinical score

**Fig 4:**
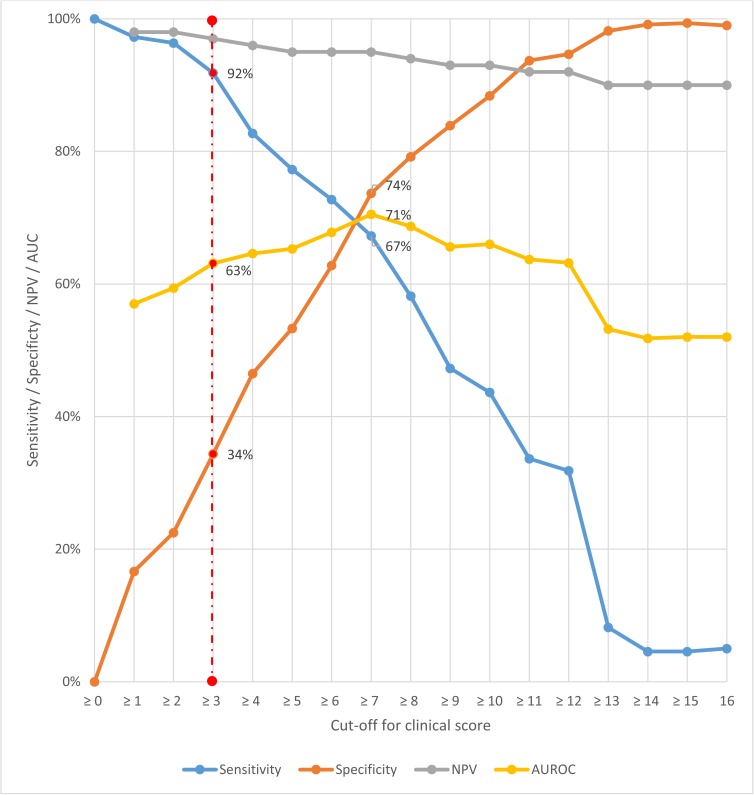
Performance of clinical score at different cut-offs offs (derivation and validation datasets combined; N = 1048). NPV = negative predictive value. AUROC = area under the receiver-operating characteristic curve.

Fig 4 shows the performance of the clinical score at different cut-offs, in terms of sensitivity, specificity, negative predictive value and AUROC in the entire dataset. A cut-off of clinical score of ≥ 3 to trigger TB investigation had sensitivity of 91.8% (95% CI 85, 96.2), specificity 34.3% (95% CI 31.3, 37.5), negative predictive value 97.3% (94.9, 98.7) and AUROC 63.1% (95% CI 60.1, 66.1). Increasing the cut-off to ≥7, where sensitivity and specificity were closest offered the best discrimination (AUROC 70.1% [95% CI 65.8, 75.1]), with improvement in specificity to 73.7% (95% CI 70.7, 76.5), but sensitivity was only 67.3% (95% CI 57.7, 75.9) although negative predictive value was maintained at 95% (95% CI 93.2, 96.5).

We selected a cut-off of clinical score ≥ 3 to trigger TB investigation as we deemed that in this population, in order to avoid missing TB diagnoses, maintaining a higher sensitivity was more important than optimising discrimination. Investigating patients who had a clinical score of ≥ 3 would have resulted in no further investigation of 30% (155/515) patients in the derivation dataset, and missed 6% (3/52) of TB diagnoses. The same cut-off for investigation in the validation dataset would have resulted in no further investigation of 33% (176/533) patients and missed 10% (6/58) of TB diagnoses. Amongst the nine patients with clinical score < 3 and TB diagnosed (4 confirmed TB, 5 clinical TB) all had had been on ART for ≥ 3 months and all reported only one symptom which was cough; median BMI was 24.3 kg/m^2^ (range 20.3–30.8) and median CD4 was 429 cells/mm^3^ (range 241–1183).

**Fig 5:**
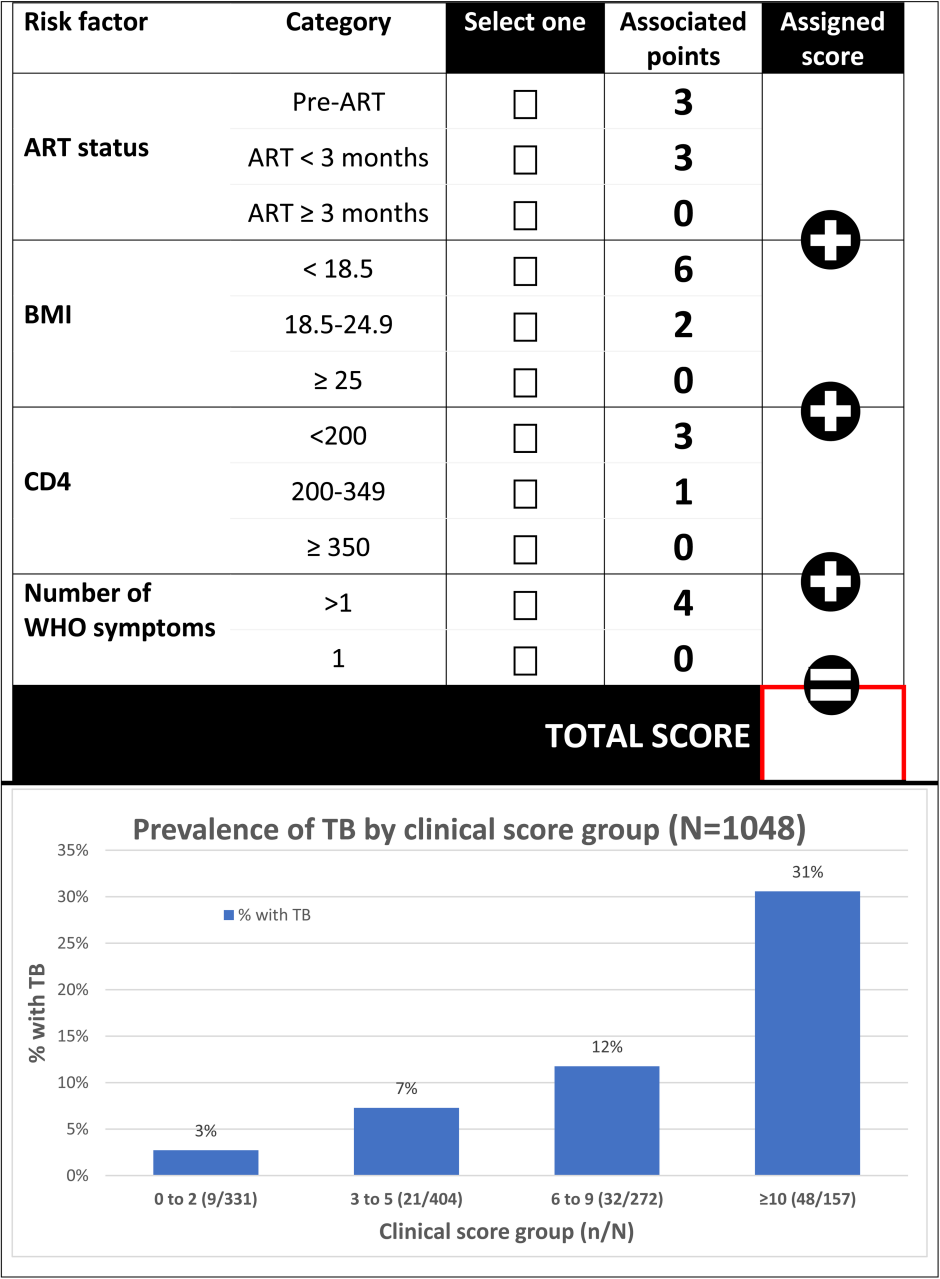
Clinical score and prevalence of TB.

Fig 5 presents a proforma of how this scoring system, using combined data from both derivation and validation datasets to demonstrate the prevalence of TB by clinical score group, could be used in practice (combined data sets, N = 1048). If investigation was prioritised for individuals with a score of ≥3, 68% (717/1048) of symptomatic individuals would be tested, among whom the prevalence of TB would be 14.1% (101/717). 32% (331/1048) of tests would be avoided using this strategy, at the cost of missing 8% (9/110) individuals with TB or 3% (9/331) with TB amongst those not tested.

## Discussion

Our study is the first to derive and internally validate a clinical score for patients attending for routine HIV care, both ART-experienced and pre-ART, for use as a second step after TB screening with the WHO tool. The score is designed to assist health care workers in resource limited settings to identify whom to prioritise for TB investigation. Our score uses elements which should be readily available at any level of health care and is simple to use, highlighting to less experienced clinicians those at greatest risk of TB, and providing a useful tool for other cadres of health care worker. In our study population, not investigating those who have a clinical score <3, amongst whom the prevalence of TB is 3% (9/331), would avoid investigation of 32% (331/1048) of those reporting WHO symptom(s), whilst missing only 8% (9/110) of TB diagnoses. We hypothesise that the WHO tool positive patients with clinical score <3 who had TB diagnoses missed were more likely to have less advanced disease and more favourable prognosis. This is suggested by their clinical characteristics (all on ART with normal weight and CD4 count >240 cells/mm^3^), and consistent with findings from other studies. [32–34] In the broader context of the original XPHACTOR study population, not investigating the 2227 who were WHO tool negative at enrolment would have missed the 28 TB diagnoses in this group (Fig 2). The overall risk of TB in those who were WHO tool negative or had clinical score <3 was 1% (37/2558), and the two step strategy (WHO tool followed by clinical score) would have avoided investigating 78% (2558/3275) of clinic attendees (Fig 2). The TB diagnoses missed using this strategy, 27% (37/138) of TB diagnoses, were mainly amongst those who were WHO tool negative.

Our clinical scoring system compares favourably, in terms of simplicity and ability to identify patients with lowest prevalence of TB, with that derived by Balcha et al, also as a second step after WHO symptom screen, for ART naïve patients attending for HIV care in Ethiopia. [8] In this smaller and as yet unvalidated (internally or externally) study, amongst 569 WHO tool positive patients, a more complex score which included Karnofsky status, MUAC, peripheral lymphadenopathy and anaemia, using a cut off of ≥2 was able to avoid investigation of 45% (255/569) of whom 8% (20/255) had culture confirmed TB. Rudolf et al derived TBscore II from a population in Bissau who were seeking care for symptoms suggestive of TB, of whom only 164 were HIV-positive. [9] Their score is also more complex than our score, incorporating physical signs in addition to symptoms and also requires both internal and external validation.[9]

The majority of our study population were established on ART in contrast to those in the meta-analysis which derived the WHO tool who were largely pre-ART, [2] and the populations used to derive clinical scores by Balcha [8] and Rudolf. [9] Thus our study addresses a key question concerning operationalisation of TB screening among the increasingly large population of adults on ART. Our study participants were established in HIV care and thus likely had had previous screening, which is known to reduce sensitivity of the WHO tool for bacteriologically confirmed TB, [2] as also is ART use. [35] Rangaka et al evaluated the utility of the WHO tool to rule out TB prior to IPT in a population similar to ours, i.e. both pre-ART and on ART although duration on ART was shorter (median 12 months), against a gold standard of culture-confirmed TB. [29] Their study suggested that amongst those on ART addition of BMI and CD4 to the tool could be considered, but recommended sputum culture first for all prior to IPT. [29] We ensured that in our clinical score we included BMI, CD4 and a measure of ART status which incorporated duration on ART, and believe that our score therefore will prove useful for all patients screened for TB during routine HIV care. Our score obviates the need for a separate tool for those pre-ART vs. on ART, although people with newly diagnosed HIV have such a high TB prevalence that investigation for all may be justified. [36]

In contrast with other studies deriving clinical algorithms or evaluating performance of the WHO rule, [2, 8, 29, 33, 35] our case definition for TB included clinical TB. This reflects the real life scenario of high TB burden resource-limited settings, and is a strength of our study. Most of our TB diagnoses were bacteriologically-confirmed pulmonary TB, which is what the WHO tool was largely designed to rule out prior to provision of IPT. [2] In sensitivity analysis restricted to bacteriologically-confirmed TB we obtained the same final multivariable model (S3 Table).

We assumed that all participants starting TB treatment or with a sample which was bacteriologically confirmed collected within six months of enrolment were likely to have had active TB at enrolment. We based this decision on data from a community survey and TB notification data in Zimbabwe, estimating a mean duration of smear-positivity prior to TB diagnosis amongst HIV-positive adults of 18–33 weeks. [11] In actual fact 90% of our study participants who started TB treatment commenced within four months of enrolment. In the derivation vs. validation dataset the interquartile range for time from enrolment to TB diagnosis is shorter (0–31 vs. 0–83 days) and this may reflect implementation of a substudy later in the course of XPHACTOR evaluating causes for persistent TB symptoms in patients without TB diagnosis by the 3-month visit. There were 47 participants (with 10 TB diagnoses) in this substudy in the validation dataset compared with 7 in the prediction dataset (with 1 TB diagnosis). We undertook the majority of our case notes reviews towards the end of the XPHACTOR study and this is reflected in the longer duration of follow up in the derivation vs. validation dataset, which may have resulted in ascertainment bias in terms of TB diagnoses made in the derivation dataset, although the total number of TB diagnoses was similar in both groups. Differences between the derivation and validation datasets represent a strength in terms of evaluating our predictive model, as non-random splitting which reduces the similarity of the two datasets is preferred for internal validation.[6]

We developed our score in accordance with TRIPOD recommendations [6] and internal validation of our final multivariable model (model A) demonstrated adequate calibration and discrimination in our validation dataset. The multivariable model resulting from our categorisation of BMI and CD4 in a clinically meaningful manner, also showed acceptable and clinically useful discrimination in the validation dataset. Our model requires external validation in order to confirm that it predicts well in individuals outside of our dataset [37] and, following this, impact studies to assess patient outcomes and cost effectiveness of this strategy. [38] Assuming external validity, our suggested threshold for investigation (clinical score ≥3), could be varied depending on available resources. We have suggestions for updating our prediction model, which we were unable to evaluate due to insufficient data: MUAC, which is simpler to measure than BMI; haemoglobin, because anaemia is a strong independent predictor of TB amongst those poised to initiate ART; [39] and viral load. [19] Recent WHO guidelines recommend ART initiation for all PLHIV at any CD4 count suggesting that in settings where viral load monitoring can be assured that CD4 count for monitoring purposes may be reduced or stopped. [40] CD4 count itself is not always easily available, and in these settings viral load monitoring is also unlikely to be easily available, but given this new guidance models without CD4 should be considered.

Strengths of our study include systematic evaluation of a representative sample of adults attending for routine HIV care who underwent rigorous assessment for TB and longitudinal follow-up which minimised the number of TB diagnoses missed, and model development and validation in accordance with TRIPOD guidelines. [6]

## Conclusions

We have developed and internally validated a simple clinical score comprising ART status, BMI, CD4 count and number of WHO symptoms, for patients attending for routine HIV care in resource limited settings. Our score is designed to identify, amongst those reporting WHO tool symptom(s), whom should be prioritised for TB investigation. Our findings are highly relevant given the national roll out of Xpert MTB/RIF in South Africa.

## Supporting information

S1 Table Characteristics of eligible participants and missing values (N = 1065). (PDF)

S2 Table Hosmer-Lemeshow test for calibration of final model (model A). (PDF)

S3 Table Model A Multivariable logistic regression analysis in derivation dataset after exclusion of all clinical TB (N = 499). (PDF)

S4 Table Model B: Multivariable logistic regression analysis in derivation dataset (N = 515). (PDF)

S1 Fig Boxplot illustrating distribution of clinical score in individuals with and without TB. (PDF)

## References

[1] World Health Organization. WHO policy on collaborative TB/HIV activities: guidelines for national programmes and other stakeholders 2012 [1st May 2013]. Available from: http://www.who.int/tb/publications/2012/tb_hiv_policy_9789241503006/en/. 23586124

[2] Getahun H, Kittikraisak W, Heilig CM, Corbett EL, Ayles H, Cain KP, et al Development of a standardized screening rule for tuberculosis in people living with HIV in resource-constrained settings: individual participant data meta-analysis of observational studies. PLoS Med. 2011;8(1):e1000391 doi: 10.1371/journal.pmed.1000391 21267059 10.1371/journal.pmed.1000391 PMC3022524

[3] Shisana O, Rehle T, LC S, Zuma K, Jooste S, N. Z, et al South African National HIV Prevalence, Incidence and Behaviour Survey, 2012 Cape Town: HSRC Press; 2014 [17th April 2015]. Available from: http://www.hsrc.ac.za/uploads/pageContent/4565/SABSSM%20IV%20LEO%20final.pdf.

[4] World Health Organization. Global tuberculosis report 2014 [17th April 2015]. Available from: http://www.who.int/tb/publications/global_report/en/.

[5] Department of Health—Republic of South Africa. National Tuberculosis Management Guidelines 2014 [2nd April 2015]. Available from: http://www.doh.gov.za/docs/hivAids/NationalTBManagementGuidelines.pdf.

[6] Moons KG, Altman DG, Reitsma JB, Ioannidis JP, Macaskill P, Steyerberg EW, et al Transparent Reporting of a multivariable prediction model for Individual Prognosis or Diagnosis (TRIPOD): explanation and elaboration. Annals of internal medicine. 2015 1 6;162(1):W1–73 doi: 10.7326/M14-0698 25560730 10.7326/M14-0698

[7] Collins GS, Reitsma JB, Altman DG, Moons KG. Transparent reporting of a multivariable prediction model for individual prognosis or diagnosis (TRIPOD): the TRIPOD statement. BMJ. 2015;350:g7594 doi: 10.1136/bmj.g7594 25569120 10.1136/bmj.g7594

[8] Balcha TT, Skogmar S, Sturegard E, Schon T, Winqvist N, Reepalu A, et al A Clinical Scoring Algorithm for Determination of the Risk of Tuberculosis in HIV-Infected Adults: A Cohort Study Performed at Ethiopian Health Centers. Open forum infectious diseases. 2014 12;1(3):ofu095 doi: 10.1093/ofid/ofu095 25734163 10.1093/ofid/ofu095 PMC4324227

[9] Rudolf F, Haraldsdottir TL, Mendes MS, Wagner AJ, Gomes VF, Aaby P, et al Can tuberculosis case finding among health-care seeking adults be improved? Observations from Bissau. Int J Tuberc Lung Dis. 2014 3;18(3):277–85 doi: 10.5588/ijtld.13.0517 24670561 10.5588/ijtld.13.0517

[10] Altman DG, Vergouwe Y, Royston P, Moons KG. Prognosis and prognostic research: validating a prognostic model. BMJ. 2009;338:b605 doi: 10.1136/bmj.b605 19477892 10.1136/bmj.b605

[11] Corbett EL, Bandason T, Cheung YB, Makamure B, Dauya E, Munyati SS, et al Prevalent infectious tuberculosis in Harare, Zimbabwe: burden, risk factors and implications for control. Int J Tuberc Lung Dis. 2009 10;13(10):1231–7 19793427 PMC3374846

[12] World Health Organization. Definitions and reporting framework for tuberculosis– 2013 revision (updated December 2014) 2013 [20th April 2015]. Available from: http://apps.who.int/iris/bitstream/10665/79199/1/9789241505345_eng.pdf?ua=1.

[13] Royston P, Moons KG, Altman DG, Vergouwe Y. Prognosis and prognostic research: Developing a prognostic model. BMJ. 2009;338:b604 doi: 10.1136/bmj.b604 19336487 10.1136/bmj.b604

[14] Steyerberg EW. Clinical Prediction Models: A Practical Approach to Development, Validatin, and updating. New York: Springer; 2009.

[15] Hanrahan CF, Golub JE, Mohapi L, Tshabangu N, Modisenyane T, Chaisson RE, et al Body mass index and risk of tuberculosis and death. AIDS. 2010 6 19;24(10):1501–8 doi: 10.1097/QAD.0b013e32833a2a4a 20505496 10.1097/QAD.0b013e32833a2a4a PMC3063388

[16] Hermans SM, Kiragga AN, Schaefer P, Kambugu A, Hoepelman AI, Manabe YC. Incident tuberculosis during antiretroviral therapy contributes to suboptimal immune reconstitution in a large urban HIV clinic in sub-Saharan Africa. PLoS One. 2010;5(5):e10527 doi: 10.1371/journal.pone.0010527 20479873 10.1371/journal.pone.0010527 PMC2866328

[17] Kibret KT, Yalew AW, Belaineh BG, Asres MM. Determinant factors associated with occurrence of tuberculosis among adult people living with HIV after antiretroviral treatment initiation in Addis Ababa, Ethiopia: a case control study. PLoS One. 2013;8(5):e64488 doi: 10.1371/journal.pone.0064488 23762214 10.1371/journal.pone.0064488 PMC3660598

[18] Kufa T, Mngomezulu V, Charalambous S, Hanifa Y, Fielding K, Grant AD, et al Undiagnosed tuberculosis among HIV clinic attendees: association with antiretroviral therapy and implications for intensified case finding, isoniazid preventive therapy, and infection control. J Acquir Immune Defic Syndr. 2012 6 1;60(2):e22–8 doi: 10.1097/QAI.0b013e318251ae0b 22627184 10.1097/QAI.0b013e318251ae0b

[19] Lawn SD, Brooks SV, Kranzer K, Nicol MP, Whitelaw A, Vogt M, et al Screening for HIV-Associated Tuberculosis and Rifampicin Resistance before Antiretroviral Therapy Using the Xpert MTB/RIF Assay: A Prospective Study. PLoS Med. 2011 7;8(7):e1001067 doi: 10.1371/journal.pmed.1001067 21818180 10.1371/journal.pmed.1001067 PMC3144215

[20] Liu E, Makubi A, Drain P, Spiegelman D, Sando D, Li N, et al Tuberculosis incidence rate and risk factors among HIV-infected adults with access to antiretroviral therapy. AIDS. 2015 7 17;29(11):1391–9 doi: 10.1097/QAD.0000000000000705 26091295 10.1097/QAD.0000000000000705 PMC4576970

[21] Nicholas S, Sabapathy K, Ferreyra C, Varaine F, Pujades-Rodriguez M, Frontieres AWGoMS. Incidence of tuberculosis in HIV-infected patients before and after starting combined antiretroviral therapy in 8 sub-Saharan African HIV programs. J Acquir Immune Defic Syndr. 2011 8 1;57(4):311–8 doi: 10.1097/QAI.0b013e318218a713 21423023 10.1097/QAI.0b013e318218a713

[22] Peck RN, Luhanga A, Kalluvya S, Todd J, Lugoba S, Fitzgerald DW, et al Predictors of tuberculosis in first 6 months after initiation of antiretroviral therapy: a case-control study. Int J Tuberc Lung Dis. 2012 8;16(8):1047–51 doi: 10.5588/ijtld.11.0772 22691942 10.5588/ijtld.11.0772 PMC3631349

[23] Van Rie A, Westreich D, Sanne I. Tuberculosis in patients receiving antiretroviral treatment: incidence, risk factors, and prevention strategies. J Acquir Immune Defic Syndr. 2011 4;56(4):349–55 doi: 10.1097/QAI.0b013e3181f9fb39 20926954 10.1097/QAI.0b013e3181f9fb39 PMC3319435

[24] Amoakwa K, Martinson NA, Moulton LH, Barnes GL, Msandiwa R, Chaisson RE. Risk factors for developing active tuberculosis after the treatment of latent tuberculosis in adults infected with human immunodeficiency virus. Open forum infectious diseases. 2015 1;2(1):ofu120 doi: 10.1093/ofid/ofu120 26034751 10.1093/ofid/ofu120 PMC4438881

[25] Ku NS, Choi YH, Kim YK, Choi JP, Kim JM, Choi JY. Incidence of and risk factors for active tuberculosis in human immunodeficiency virus-infected patients in South Korea. Int J Tuberc Lung Dis. 2013 6;17(6):777–81 doi: 10.5588/ijtld.12.0607 23676161 10.5588/ijtld.12.0607

[26] Corbett EL, Churchyard GJ, Clayton TC, Williams BG, Mulder D, Hayes RJ, et al HIV infection and silicosis: the impact of two potent risk factors on the incidence of mycobacterial disease in South African miners. AIDS. 2000 12 1;14(17):2759–68 11125895 10.1097/00002030-200012010-00016

[27] Claassens MM, van Schalkwyk C, du Toit E, Roest E, Lombard CJ, Enarson DA, et al Tuberculosis in healthcare workers and infection control measures at primary healthcare facilities in South Africa. PLoS One. 2013;8(10):e76272 doi: 10.1371/journal.pone.0076272 24098461 10.1371/journal.pone.0076272 PMC3788748

[28] Baussano I, Williams BG, Nunn P, Beggiato M, Fedeli U, Scano F. Tuberculosis incidence in prisons: a systematic review. PLoS Med. 2010;7(12):e1000381 doi: 10.1371/journal.pmed.1000381 21203587 10.1371/journal.pmed.1000381 PMC3006353

[29] Rangaka MX, Wilkinson RJ, Glynn JR, Boulle A, van Cutsem G, Goliath R, et al Effect of antiretroviral therapy on the diagnostic accuracy of symptom screening for intensified tuberculosis case finding in a South African HIV clinic. Clin Infect Dis. 2012 12;55(12):1698–706 doi: 10.1093/cid/cis775 22955441 10.1093/cid/cis775 PMC3501332

[30] Hosmer DW, Lemeshow S, Sturdivant RX. Applied Logistic Regression, Third Edition John Wiley & Sons; 2013.

[31] Gupta RK, Lawn SD, Bekker LG, Caldwell J, Kaplan R, Wood R. Impact of human immunodeficiency virus and CD4 count on tuberculosis diagnosis: analysis of city-wide data from Cape Town, South Africa. Int J Tuberc Lung Dis. 2013 8;17(8):1014–22 doi: 10.5588/ijtld.13.0032 23827024 10.5588/ijtld.13.0032 PMC3990260

[32] Balcha TT, Sturegard E, Winqvist N, Skogmar S, Reepalu A, Jemal ZH, et al Intensified tuberculosis case-finding in HIV-positive adults managed at Ethiopian health centers: diagnostic yield of Xpert MTB/RIF compared with smear microscopy and liquid culture. PLoS One. 2014;9(1):e85478 doi: 10.1371/journal.pone.0085478 24465572 10.1371/journal.pone.0085478 PMC3899028

[33] Cain KP, McCarthy KD, Heilig CM, Monkongdee P, Tasaneeyapan T, Kanara N, et al An algorithm for tuberculosis screening and diagnosis in people with HIV. N Engl J Med. 2010 2 25;362(8):707–16 doi: 10.1056/NEJMoa0907488 20181972 10.1056/NEJMoa0907488

[34] Lawn SD, Kerkhoff AD, Vogt M, Ghebrekristos Y, Whitelaw A, Wood R. Characteristics and early outcomes of patients with Xpert MTB/RIF-negative pulmonary tuberculosis diagnosed during screening before antiretroviral therapy. Clin Infect Dis. 2012 4;54(8):1071–9 doi: 10.1093/cid/cir1039 22318975 10.1093/cid/cir1039 PMC3309885

[35] Ahmad Khan F, Verkuijl S, Parrish A, Chikwava F, Ntumy R, El-Sadr W, et al Performance of symptom-based tuberculosis screening among people living with HIV: not as great as hoped. AIDS. 2014 6 19;28(10):1463–72 doi: 10.1097/QAD.0000000000000278 24681417 10.1097/QAD.0000000000000278 PMC5116236

[36] Ndlovu N, Chihota V, Hanifa Y, Fielding K, Grant A, Maesela C, et al., editors. Routine testing with Xpert MTB/RIF for people testing HIV positive at antenatal clinics and HIV counselling and testing. Abstract (A2638640) 4th SA TB Conference; 2014 Durban, South Africa.

[37] Moons KG, Kengne AP, Grobbee DE, Royston P, Vergouwe Y, Altman DG, et al Risk prediction models: II. External validation, model updating, and impact assessment. Heart. 2012 5;98(9):691–8 doi: 10.1136/heartjnl-2011-301247 22397946 10.1136/heartjnl-2011-301247

[38] Moons KG, Altman DG, Vergouwe Y, Royston P. Prognosis and prognostic research: application and impact of prognostic models in clinical practice. BMJ. 2009;338:b606 doi: 10.1136/bmj.b606 19502216 10.1136/bmj.b606

[39] Kerkhoff AD, Wood R, Cobelens FG, Gupta-Wright A, Bekker LG, Lawn SD. Resolution of anaemia in a cohort of HIV-infected patients with a high prevalence and incidence of tuberculosis receiving antiretroviral therapy in South Africa. BMC Infect Dis. 2014;14:3860 doi: 10.1186/s12879-014-0702-1 25528467 10.1186/s12879-014-0702-1 PMC4300078

[40] World Health Organization. Guideline on when to start antiretroviral therapy and on pre-exposure prophylaxis for HIV 2015 [28th April 2016]. Available from: http://www.who.int/hiv/pub/guidelines/earlyrelease-arv/en/. 26598776

