# Supplementary material for: A clinical scoring system to prioritise investigation for tuberculosis among adults attending HIV clinics in South Africa
Source: PLoS One. 2017 Aug 3;12(8):e0181519. doi: 10.1371/journal.pone.0181519 (PMC5542442; doi:10.1371/journal.pone.0181519)
Supplement: S1 Table — (PDF) [file pone.0181519.s001.pdf]

S1 Table. Characteristics of eligible participants and missing values (N=1065)

**Characteristic**		Derivation dataset (N=525)		Validation dataset (N=540)	
		Value<br><br>N (%)	Missing values N (%)	Value<br><br>N (%)	Missing values N (%)
**Demographics**					
**Age, years**	Median (IQR)	41 (34,48)	0	41 (34,48)	0
**Sex**	Female	353 (67.2)	0	382 (70.7)	0
**Alcohol history**	Never [1]	315 (60)	0	360 (66.7)	0
**Smoking history**	Never [2]	361 (68.8)	0	389 (72.0)	0
**HIV/TB history**					
**Participant category**	On ART [3]	377 (71.8)	0	387 (71.7)	0
**Duration since HIV diagnosed, months**	Median (IQR)	56 (21,95)	10 (2)	51 (6,97)	2 (0.4)
**Duration on ART, months**	Median (IQR)	55 (26,85)	1/377(0.3)	51 (28,83)	0
**Ever had IPT**	Yes	51 (9.7)	0	19 (3.5)	0
**Ever had CPT**	Yes	378 (72.0)	0	360 (66.7)	0
**Previous TB treatment**	Yes	205 (39.1)	0	202 (37.4)	0
**WHO symptoms at enrolment**					
	Cough	308 (58.7)	0	354 (65.6)	0
	Weight loss	238/524(45.4)	1 (0.2)	224 (41.5)	0
	Night sweats	133 (25.3)	0	132 (24.4)	0
	Fever	99 (18.9)	0	89 (16.5)	0
**Number of symptoms**		1 (1,2)	0	1 (1,2)	0
**Duration of WHO symptoms[4], days**	Median (IQR)	30 (8,94)	5 (1)	28 (7,84)	4 (0.7)
**CD4 / BMI at enrolment**					
**CD4, cells/mm^3**	Median (IQR)	379 (228,543)	2 (0.4)	335(168,559)	1 (0.2)
**Time from CD4 to enrolment, days**	Median (IQR)	147 (43,259)	2 (0.4)	118 (27,267)	6 (1)
**BMI, kg/m^2**	Median (IQR)	24.0(20.6,28.5)	1 (0.2)	24.1(20.3,28.4)	2 (0.4)
**TB diagnoses**					
	Total	52 (9.9)	0	60 (11.1)	0
	Confirmed TB	36 (6.9)	0	41 (7.6)	0
	Clinical TB	16 (3.1)	0	19 (3.5)	0
**Time from enrolment to TB diagnosis [5], days**	Median (IQR)	7 (0,31)	0	13 (0,83)	1 (0.2)
**Follow up**					
**Time from enrolment to most recent of last study / clinic [6] visit, days**	Median (IQR)	281 (203,347)	1 (0.2)	181 (133,231)	1 (0.2)
**Alive 6 months after enrolment [7]**	Yes	487 (98) (N=497)	28 (5.3)	469 (98) (N=479)	61(11.3)

[1] compared with any alcohol in last 1 year; [2] compared with ever/ex-smoker; [3] compared with pre-ART group; [4] duration WHO tool positive;[5] defined as earliest of positive TB test or date TB treatment started; [6] Most recent clinic visit at time of clinic file review;
[7] Amongst participants with most recent study/clinic visit <6 months from enrolment, if participant had valid South African ID number and demise not reported by Department of home affairs / participant-nominated contacts / clinic staff within 6 months of enrolment, participant assumed to be alive at 6 months after enrolment.

IPT=isoniazid preventive therapy; CPT=cotrimoxazole preventive therapy
