# Supplementary material for: A clinical scoring system to prioritise investigation for tuberculosis among adults attending HIV clinics in South Africa
Source: PLoS One. 2017 Aug 3;12(8):e0181519. doi: 10.1371/journal.pone.0181519 (PMC5542442; doi:10.1371/journal.pone.0181519)
Supplement: S2 Table — (PDF) [file pone.0181519.s002.pdf]

S2 Table. Hosmer-Lemeshow test for calibration of final model (model A)

	**Derivation dataset [1]**				**Validation dataset [2]**			
			**TB**				**TB**	
**Decile**	***N***	**Cut off[3]**	**Observed**	**Predicted**	***N***	**Cut off[3]**	**Observed**	**Predicted**
**1**	52	0.0126	0	0.4	54	0.0098	1	0.3
**2**	51	0.0220	2	0.9	53	0.0186	1	0.7
**3**	52	0.0308	0	1.4	53	0.0288	2	1.3
**4**	51	0.0448	2	1.9	54	0.0450	4	2.0
**5**	52	0.0611	2	2.7	53	0.0627	4	2.9
**6**	51	0.0805	5	3.6	53	0.1024	5	4.2
**7**	52	0.1078	7	4.9	54	0.1478	6	6.6
**8**	51	0.1604	5	6.6	53	0.2044	8	9.3
**9**	52	0.2681	12	10.6	53	0.3346	6	13.8
**10**	51	0.5963	17	19.0	53	0.6479	21	23.7
	**515**		**52**	**52**	**533**		**58**	**64.8**

[1] Hosmer-Lemeshow p=0.65

[2] Hosmer-Lemeshow p=0.31

[3] Upper boundary of predicted risk

Observed = observed number with TB

Predicted = expected number with TB predicted by model
