# Supplementary material for: A clinical scoring system to prioritise investigation for tuberculosis among adults attending HIV clinics in South Africa
Source: PLoS One. 2017 Aug 3;12(8):e0181519. doi: 10.1371/journal.pone.0181519 (PMC5542442; doi:10.1371/journal.pone.0181519)
Supplement: S3 Table — (PDF) [file pone.0181519.s003.pdf]

S3 Table: Model A Multivariable logistic regression analysis in derivation dataset after exclusion of all clinical TB (N=499)

Predictor		Patients with TB N=36/499 n/N (%)	Unadjusted odds ratio (95% CI)	P value (Wald)	Adjusted [3] odds ratio (95% CI)	P value	Adjusted β coefficient (log [adjusted OR]) (95% CI)
**Age [1], years**			1.00 (0.96, 1.03)	0.89			
**Sex**	Male	16/163 (9.8%)	1				
	Female	20/336 (6.0%)	0.59 (0.29, 1.15)	***0.12***			
**Smoking status**	Never smoked	20/346 (5.8%)	1				
	Current or ex-smoker	16/153 (10.5%)	1.90 (0.96, 3.78)	***0.07***			
**Alcohol status**	Current	15/199 (7.5%)	1				
	None in last 1 year	21/300 (7.0%)	0.92 (0.46, 1.84)	0.82			
**ART status**	On ART ≥ 3 months	18/341 (5.3%)	1		1		0
	Pre-ART / ART <3 months	18/158 (11.4%)	2.31 (1.17, 4.57)	***0.02***	1.84 (0.87, 3.89)	0.11	0.61 (-0.14, 1.36)
**Ever had CPT**	No / don't know	12/138 (8.7%)	1				
	Yes	24/361 (6.7%)	0.75 (0.36, 1.54)	0.43			
**Previous history of TB**	No	25/306 (8.2%)	1				
	Yes	11/193 (7.7%)	0.68 (0.33, 1.41)	0.30			
**Number of WHO symptoms**	1 symptom	11/334 (3.3%)	1		1		0
	> 1 symptom	25/165 (15.2%)	5.24 (2.51, 11.00)	***<0.001***	4.33 (2.02, 9.23)	***<0.001***	1.46 (0.70, 2.23)
**Duration of WHO tool symptoms**	<1 week	2/96 (2.1%)	1				
	≥ 1 week	34/403 (8.4%)	4.33 (1.02, 18.35)	***0.05***			
**BMI [1,2], kg/m^2**			0.87 (0.80, 0.94)	***0.001***	0.88 (0.81, 0.96)	***0.004***	-0.12 (-0.21, -0.04)
**CD4 [1,2], cells/mm^3**			0.997 (0.995, 0.998)	***<0.001***	0.998 (0.996, 0.999)	***0.012***	-0.002 (-0.004, -0.0005)

[1] Age, BMI and CD4 count were modelled as continuous variables

[2] In the multivariable analysis BMI and CD4 count were modelled as continuous variables, a linear relationship with the outcome was found to be adequate after modelling using fractional polynomials.

[3] Adjusted for all variables shown. 100 unit increase in CD4 corresponds to reduction in adjusted odds ratio (aOR) of TB of 0.78 (95% CI 0.64, 0.95); 5 unit increase in BMI corresponds to reduction in aOR of TB of 0.54 (95% CI 0.35, 0.82).
Intercept (log odds) for multivariable model is 0.21. In the multivariable model we found no statistically significant interaction between remaining variables and "ART status".
