# Supplementary material for: A clinical scoring system to prioritise investigation for tuberculosis among adults attending HIV clinics in South Africa
Source: PLoS One. 2017 Aug 3;12(8):e0181519. doi: 10.1371/journal.pone.0181519 (PMC5542442; doi:10.1371/journal.pone.0181519)
Supplement: S4 Table — (PDF) [file pone.0181519.s004.pdf]

S4 Table. Model B: Multivariable logistic regression analysis in derivation dataset (N=515)

**Predictor**		**Patients with TB N=52/515 n/N (%)**	**Unadjusted odds ratio (95% CI)**	**P value (Wald)**	**Adjusted [3] odds ratio Model B (95% CI)**	**P value (Wald)**	**Adjusted β coefficient (log [adjusted OR]) (95% CI)**
**Age [1], years**			1.00 (0.97, 1.03)	0.96			
**Sex**	Male	23/170 (13.5%)	1				
	Female	29/345 (8.4%)	0.59 (0.32, 1.05)	***0.07***			
**Smoking status**	Never smoked	28/354 (7.9%)	1				
	Current or ex-smoker	24/161 (14.9%)	2.04 (1.14, 3.64)	***0.02***			
**Alcohol status**	Current	23/207 (11.1%)	1				
	None in last 1 year	29/308 (9.4%)	0.83 (0.47, 1.48)	0.53			
**ART status**	On ART ≥ 3 months	24/347 (6.9%)	1		1		0
	Pre-ART / ART <3 months	28/168 (16.7%)	2.69 (1.51, 4.80)	***0.001***	2.07 (1.07, 4.01)	***0.03***	0.73 (0.06, 1.39)
**Ever had CPT**	No / don't know	19/145 (13.1%)	1				
	Yes	33/370 (8.9%)	0.65 (0.36, 1.18)	***0.16***			
**Previous history of TB**	No	33/314 (10.5%)	1				
	Yes	19/201 (9.5%)	0.89 (0.49, 1.61)	0.70			
**Cough**	No	16/211 (7.6%)	1		1		0
	Yes	36/304 (11.8%)	1.64 (0.88-3.03)	***0.12***	2.96 (1.50, 5.85)	***0.002***	1.08 (0.40, 1.77)
**Fever**	No	38/418 (9.1%)	1				
	Yes	14/97 (14.4%)	1.69 (0.87-3.25)	***0.12***			
**Night sweats**	No	31/384 (8.1%)	1		1		0
	Yes	21/131 (16.0%)	2.17 (1.20-3.94)	***0.01***	1.99 (1.02, 3.89)	***0.04***	0.69 (0.02, 1.36)
**Unintentional weight loss**	No	12/280 (4.3%)	1		1		0
	Yes	40/235 (17.0%)	4.58 (2.34-8.96)	***<0.001***	4.08 (1.96, 8.49)	***<0.001***	1.41 (0.67, 2.14)
**BMI [1,2], kg/m²**			0.88 (0.82, 0.94)	***<0.001***	0.90 (0.84, 0.97)	***0.005***	-0.10 (-0.17, -0.03)
**CD4 [1,2], cells/mm³**			0.997 (0.995, 0.998)	***<0.001***	0.997 (0.996, 0.999)	***0.009***	-0.002 (-0.004, -0.0005)

[1] Age, BMI and CD4 count were modelled as continuous variables
[2] BMI and CD4 count were modelled as continuous variables, a linear relationship with the outcome was found to be a good approximation after assessment of nonlinearity using fractional polynomials.
[3] Adjusted for all variables shown. 100 unit increase in CD4 corresponds to reduction in adjusted odds ratio (aOR) of TB of 0.81 (95% CI 0.69, 0.95); 5 unit increase in BMI corresponds to reduction in aOR of TB of 0.61 (95% CI 0.43, 0.86).
In the multivariable model we tested for interactions between "ART status" and CD4 cell count, "ART status" and BMI, "ART status" and cough, "ART status" and night sweats, "ART status" and weight loss. Interaction term with p<0.05: ART status and cough.
Intercept (log odds) for multivariable model is 0.32
In derivation vs. validation datasets: Hosmer-Lemeshow statistic p=0.81 vs. p=0.01, AUROC 0.82 (95% CI 0.76-0.88) vs. AUROC 0.75 (95% CI 0.69-0.82)
