# Supplementary material for: A clinical scoring system to prioritise investigation for tuberculosis among adults attending HIV clinics in South Africa
Source: PLoS One. 2017 Aug 3;12(8):e0181519. doi: 10.1371/journal.pone.0181519 (PMC5542442; doi:10.1371/journal.pone.0181519)

S1 Fig. Boxplot illustrating distribution of clinical score in individuals with and without TB

N=515 in derivation dataset with 52 TB diagnoses; N=535 in validation dataset with 58 TB diagnoses

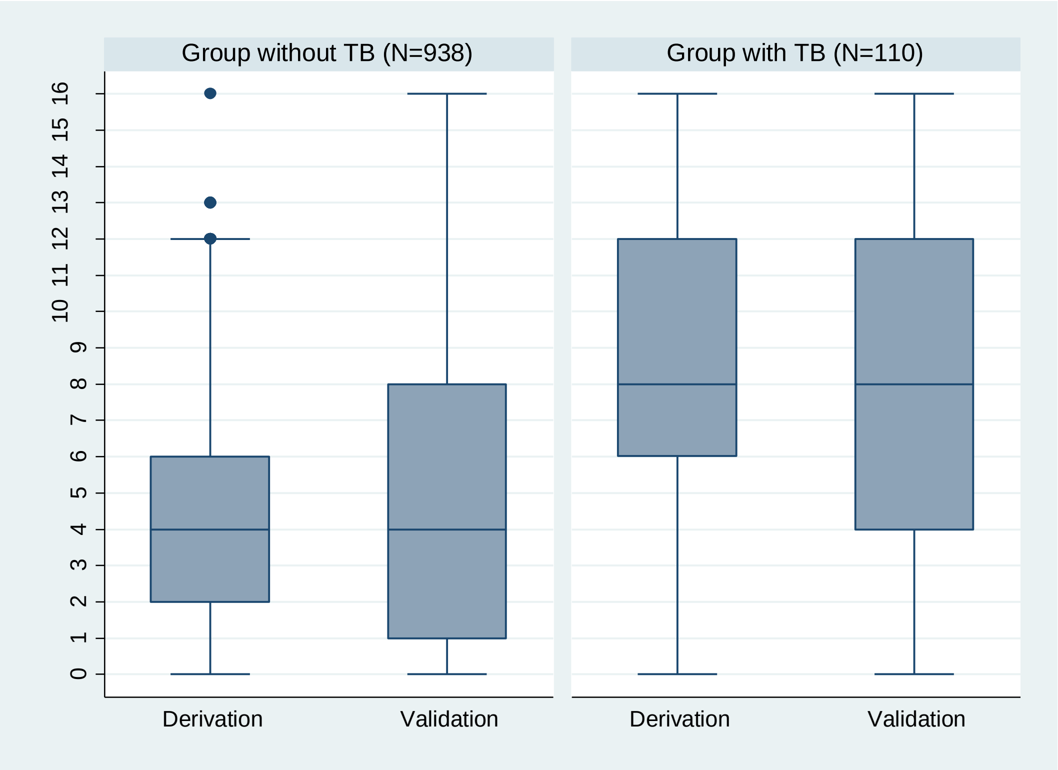

Supplement: S1 Fig — (PDF) [file pone.0181519.s005.pdf]
